# Gene Therapy for Dopamine Dyshomeostasis: From Parkinson's to Primary Neurotransmitter Diseases

**DOI:** 10.1002/mds.29416

**Published:** 2023-05-05

**Authors:** Joanne Ng, Serena Barral, Simon N. Waddington, Manju A. Kurian

**Affiliations:** ^1^ Gene Transfer Technology Group, EGA‐Institute for Women's Health University College London London United Kingdom; ^2^ Genetic Therapy Accelerator Centre, Department of Neurodegenerative Disease, Queen Square Institute of Neurology University College London London United Kingdom; ^3^ Developmental Neurosciences, Zayed Centre for Research into Rare Disease in Children, GOS‐Institute of Child Health University College London London United Kingdom; ^4^ Wits/SAMRC Antiviral Gene Therapy Research Unit, Faculty of Health Sciences University of the Witwatersrand Johannesburg South Africa; ^5^ Department of Neurology Great Ormond Street Hospital for Children London United Kingdom

**Keywords:** gene therapy, AAV, dopamine transporter deficiency syndrome, AADC deficiency, Parkinson's

## Abstract

Neurological disorders encompass a broad range of neurodegenerative and neurodevelopmental diseases that are complex and almost universally without disease modifying treatments. There is, therefore, significant unmet clinical need to develop novel therapeutic strategies for these patients. Viral gene therapies are a promising approach, where gene delivery is achieved through viral vectors such as adeno‐associated virus and lentivirus. The clinical efficacy of such gene therapies has already been observed in two neurological disorders of pediatric onset; for spinal muscular atrophy and aromatic L‐amino acid decarboxylase (AADC) deficiency, gene therapy has significantly modified the natural history of disease in these life‐limiting neurological disorders. Here, we review recent advances in gene therapy, focused on the targeted delivery of dopaminergic genes for Parkinson's disease and the primary neurotransmitter disorders, AADC deficiency and dopamine transporter deficiency syndrome (DTDS). Although recent European Medicines Agency and Medicines and Healthcare products Regulatory Agency approval of Upstaza (eladocagene exuparvovec) signifies an important landmark, numerous challenges remain. Future research will need to focus on defining the optimal therapeutic window for clinical intervention, better understanding of the duration of therapeutic efficacy, and improved brain targeting. © 2023 The Authors. *Movement Disorders* published by Wiley Periodicals LLC on behalf of International Parkinson and Movement Disorder Society.

## Introduction

Recent advances in genetic therapies for the treatment of neurological disorders comprise several approaches, including gene addition, gene silencing, genome editing, and advanced editing strategies such as prime editing. Viral vectors infect cells with high efficiency and can deliver genetic payloads such as complementary DNA (cDNA), guide‐RNAs, small interfering RNAs (siRNA) and microRNAs.^1^ For delivery of RNA therapeutics, non‐viral delivery methods are also showing great potential for a broad spectrum of applications; these range from the antisense oligonucleotide therapy, nusinersen for spinal muscular atrophy (SMA) to mRNA based coronavirus disease 2019 (Covid19) vaccinations.^2^, ^3^ An important difference of viral vector‐delivered genetic therapy is the prospect of single dosing to potentially convey life‐long genetic therapy. However in pediatric disorders especially where transgene expression potentially may wane over time, boosting expression may be desirable. Re‐administration of adeno‐associated virus (AAV) gene therapies, for example, using the same serotype has not been translated clinically, as viral vectors and potentially transgene may prime the immune system.

AAVs have been widely used for gene therapy for central nervous system (CNS) disorders. Single stranded AAV can package ~4.7 kb DNA. AAVs can infect a wide range of mitotic and post‐mitotic cell types.^4^ The current consensus is that AAV does not cause human disease and AAV vectors are considered non‐pathogenic.^4^ Recent identification of high levels of AAV serotype 2 in cases of unexplained acute pediatric hepatitis have been reported.^5^ Although these findings do not prove causality, further studies are required to better understand this association. Wild‐type AAV integrates into the host genome, notably at the *AAVS1* site on human chromosome 19, via a Rep‐dependent mechanism to establish latency.^4^ AAV vectors, which are devoid of Rep, were thought to exist episomally, but there is evidence of random host integration in preclinical AAV gene therapy studies.^6^ Recombinant AAVs (rAAV) used in viral gene therapy are composed of the same capsid sequence and structure as wild‐type AAVs, but their genomes are devoid of all AAV protein‐coding sequences with therapeutic gene expression cassettes designed in their place. The only sequences of viral origin are the inverted terminal repeats (ITRs) required to guide genome replication and packaging during vector production.^4^

There are several naturally occurring AAV serotypes that display different tissue tropisms that lend themselves suitable for different disorders.^7^ The AAV2 and AAV9 serotypes have excellent tropism for neurons with high durability of AAV episomes residing in the nucleus of transduced cells.^7^ Two AAV based gene therapies have received United States Federal Drug Administration (FDA), European Medicines Agency (EMA), and Medicines and Healthcare products Regulatory Agency (MHRA) approval: Luxturna (Avoretigene neparvovec‐rzyl AAV2 for retinitis pigmentosa) and Zolgensma (onasemnogene abeparvovec‐xioi AAV9 for SMA). More recently Upstaza (eladocagene exuparvovec AAV2 for aromatic L‐amino acid decarboxylase [AADC] deficiency) has received EMA and MHRA approval. Capsid engineering approaches have been developed to enhance specific transduction properties for enhanced liver or CNS transduction. Synthetic capsids such as AAV PHPB and MNM008 have shown superior neuronal transduction efficiencies to AAV2 and AAV9, but have to date, only been tested in preclinical settings.^8^, ^9^

Lentivirus vectors (LV) are predominantly based on HIV‐1 of the retrovirus family, and are capable of infecting both mitotic and post‐mitotic cells. These vectors have larger packaging capacity of approximately 11 kb of single stranded RNA with long‐term transgene expression.^10^ A concern for the clinical use of LV has been the possibility of integration into the host genome although rigorous design modifications have allayed these concerns. This has led to the development of integration‐defective lentiviral vectors that have shown promise in preclinical studies.^11^, ^12^

Both AAV and lentiviral gene therapies have been tested in clinical trial for idiopathic Parkinson's disease (PD). PD is a progressive neurogenerative disorder characterized by bradykinesia, hypomimia, tremor, muscle rigidity, stooped posture, postural instability, and shuffling gait, as well as a number of non‐motor features. It is the second most common neurodegenerative disorder affecting 6 million people worldwide.^13^ Although the movement disorder is often the first symptom patients present to the clinician, the disorder is associated with complex non‐motor symptoms including postural hypotension, constipation, and neuropsychiatric symptoms of depression, dopamine dysregulation symptoms of impaired impulse control, and Lewy body dementia.

The focus of PD treatments includes pharmacotherapeutic strategies available for the management of motor symptoms, but none are truly disease‐modifying. Medications, such as levodopa (l‐dopa) and other dopaminergic medications can be highly beneficial initially, but the therapeutic response declines with time, with debilitating fluctuations between on and off states and intolerable drug‐related dyskinesia. Deep brain stimulation (DBS) surgery is an option and may be indicated in those whose response to medication is unstable, but is not a disease modifying treatment. The potential for targeted gene therapy renders PD a suitable disease for this approach, given that affected dopamine synthesizing neurons are specifically located in the substantia nigra (SN), although the motor control network, including limbic, midbrain, and brainstem neurons are still subject to dysfunction and degeneration.^14^ Therefore, targeting the nigrostriatal system alone may only result in a partial benefit. Despite the uncertainty around the etiology of dopaminergic neuronal loss in PD, several gene therapy strategies have been evaluated clinically. These have focused on four main targeted approaches, namely: (1) restoring dopamine synthesis; (2) neuroprotection; (3) genetic neuromodulation; and (4) modulation of disease‐modifying variants such as pathogenic glucocerebrosidase (*GBA*) gene variants.

In this review, we summarize gene therapy advances focused on the delivery of dopaminergic genes for PD and the primary neurotransmitter disorders, AADC deficiency, and dopamine transporter deficiency syndrome (DTDS). Future directions, to develop next generation gene therapies for dopaminergic disorders, are also discussed.

## Viral Gene Therapy Approaches in PD

PD is pathologically characterized by the gradual loss of dopaminergic neurons in the substantia nigra pars compacta (SNpc); this results in dopamine depletion in the motor regions of the putamen. As PD progresses, there is degradation of striatal AADC with loss of dopaminergic SNpc striatal nerve terminals. To compensate for the loss of dopamine synthesis in PD, gene therapies delivering dopamine synthesis enzymes have been developed. There have been 15 published PD gene therapy trials, with gene delivery targeted by intraparenchymal stereotactic injections.^14^ These include AAV2‐GDNF (one trial),^15^ and AAV2‐NRTN (five trials, with three publications on post‐mortem data),^16^, ^17^, ^18^, ^19^, ^20^, ^21^, ^22^, ^23^ AAV2‐GAD (four trials),^24^, ^25^, ^26^, ^27^ ProSavin (one trial with one long term follow‐up study),^28^, ^29^ and AAV2‐AADC (three trials).^30^, ^31^, ^32^, ^33^, ^34^, ^35^ In these clinical trials, outcomes were reported using the Unified Parkinson's Disease Rating Scale (UPDRS)—a four part assessment that includes clinical motor examination observations (part III), motor complications (part IV), imaging, and safety through adverse events (AEs) reporting. These clinical trials demonstrate the clinical feasibility and safety of gene therapy for PD.

### Gene Therapy with *AADC* Gene

**FIG 1:**
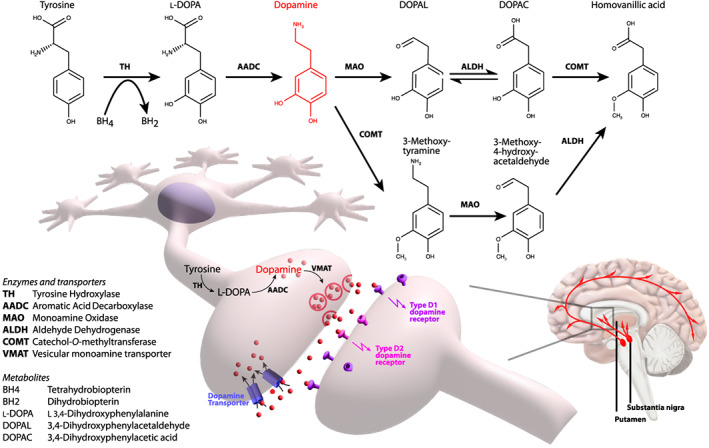
Schematic of dopamine synthesis and neurotransmission. [Color figure can be viewed at wileyonlinelibrary.com]

The current first‐line pharmacological treatment for PD is oral l‐dopa, but clinical response declines with progression of disease as more dopaminergic neurons degenerate and levels of AADC enzyme (required for the conversion of l‐dopa to dopamine) (Fig. 1) decline. To address this decline in enzyme levels, gene therapy approaches delivering *AADC* using rAAV2 have been evaluated in several phase‐I open‐label studies. To date, 31 participants have received rAAV2‐hAADC through bilateral intraputaminal infusions to express hAADC enzyme in non‐degenerating striatal neurons (Table 1).^30^, ^31^, ^32^, ^33^, ^34^, ^35^

**TABLE 1:** Clinical trials in dopamine synthesis gene therapies for PD

Study	Dose (vg) and participants	Disease duration (years)	Follow up (months)	Outcomes measures	Adverse events
Eberling et al.^30^ Open label/phase I Putaminal rAAV2.AADC	9 × 10^10^ (200 μL) n = 5	10.8 ± 7.5	6	28% total UPDRS +34% UPDRS‐III *off* state No change in UPDRS *on*‐state, *on*‐time or LED 30% increase FMT‐PET uptake	Asymptomatic intracranial hemorrhage related to surgery Transient headache and surgical site pain
Christine et al.^31^ Open label/phase I Putaminal rAAV2.AADC	9 × 10^10^ (200 μL) n = 5 3 × 10^11^ (200 μL) n = 5 Same cohort 9 × 10^10^ as Eberling et al.^30^	9.3 ± 5.3	6	31% total UPDRS and 36% UPDRS‐III *off*‐state, 32% UPDRS *on*‐state 3.1 hours reduction in *off* time 30% increase FMT‐PET uptake low dose 75% increase FMT‐PET uptake high dose	2 asymptomatic intracranial hemorrhages related to surgery 1 symptomatic intracranial hemorrhage with resolving hemiplegia and aphasia
Valles et al.^32^ Open label/phase I Putaminal rAAV2.AADC	9 × 10^10^ (200 μL) n = 5 Same cohort as Eberlin Christine et al.	9.3 ± 5.3	6	Study of accuracy of putaminal targeting Outcomes as per Christine et al.^31^	Same cohort as Eberling et al.^30^
Muramatsu et al.^33^ Open label/phase I Putaminal rAAV2.AADC	3 × 10^11^ (200 μL) n = 6	10.0 ± 4.5	6	28% total UPDRS and 45% UPDRS‐III (*off*) No change in UPDRS (*On*) or LED 56% increase FMT‐PET uptake	1 venous hemorrhage with transient arm weakness with full recovery
Mittermeyer et al.^34^ Open label/phase I Putaminal rAAV2.AADC	9 × 10^10^ (200 μL) n = 5 3 × 10^11^ (200 μL) n = 5 Same cohort as Christine et al.^31^	9.3 ± 5.3	48	Safety and tolerability of long term gene expression. UPDRS improvement that returned to presurgical values at 12 months 56% increase in FMT‐PET uptake	As per Christine et al.^31^ 4 experienced transient increase in dyskinesia
Christine et al.^35^ Open‐label/phase I Putaminal rAAV2.AADC	7.5 × 10^11^ (450 μL) n = 5 1.5 × 10^12^ (900 μL) n = 5 4.7 × 10^12^ (900 μL) n = 5	9.5 ± 0.9	36	F‐DOPA‐PET showed dose dependent increase 13% ± 7%, 56 ± 13%, 79% ± 15% at 6 months. Significantly showed stabilization or improvement in UPDRS III, reduced LED, clinical and patient global impression of improvement scores and PDQ39 (quality of life) at 12, 24, and 36 months. 1 patient in lowest dosage cohort had DBS insertion at 34 months	4 experienced transient increase in dyskinesia 1 deep vein thrombosis, atrial fibrillation and pulmonary embolus 1 small bowel obstruction
Palfi et al.^28^ Open label/phase I/II Putaminal ProSavin LV TH‐AADC‐GCH	15 1.9 × 10^7^ (n = 5) 4 × 10^7^ (n = 5) 1 × 10^8^ (n = 5)	13.9 ± 5.3	12	11.2 points UPDRS‐III (*Off*‐state) and 2 points UPDRS‐IV improvement Improvement in UPDRSII and PDQ‐39 with reduced LED No significant change in LED or F‐DOPA‐PET	54 mild/ moderate events reported including dyskinesia, tremor, anxiety, on–off phenomena
Palfi et al.^29^ Open label/phase I/II Putaminal ProSavin LV TH‐AADC‐GCH	15 1.9 × 10^7^ (n = 5) 4 × 10^7^ (n = 5) 1 × 10^8^ (n = 5)	13.9 ± 5.3	24–72	8.2 points UPDRS‐III (Off‐state) improvement Other improvements not sustained long term. 8 patients required DBS intervention after 2 years with continued disease progression	3 severe events dyskinesia, acute psychosis and nervous system disorder (not specified) 1 death at 4 years post infusion (cardiorespiratory arrest) 1 death at 6 years post infusion (peritonitis)

Abbreviations: DBS, deep brain stimulation; LED, levodopa equivalent dose; PDQ39, Parkinson's disease questionnaire 39; UPDRS, unified Parkinson's disease rating scale; vg, vector genomes.

One study found a 36% *off*‐state UPDRS‐III improvement at 6 months,^31^ followed by a gradual decline to pre‐surgical baseline at 48 months.^34^ A similar *off*‐state UPDRS‐III improvement (21% and 46% improvement) was observed in two further studies at 18–39 months, respectively, with a trend toward greater improvement at higher dose.^33^, ^35^ No significant changes in the *on*‐state UPDRS‐III scores was seen. Three studies showed improved *off*‐time of 0.6–3.1 hours per day^31^, ^33^, ^34^ and one showed an improvement in *on*‐time without dyskinesia of 1.5–3.3 hours per day.^34^ All four studies undertook post‐gene therapy fluoro‐m‐tyrosine (FMT)‐positron emission tomography (PET) analysis with a 25%–75% increase in putaminal uptake and an increasing trend with higher dosages.^30^, ^31^, ^32^, ^35^ One study reported 18Fluorodopa (18F‐DOPA) PET studies in 10 trial participants, demonstrating a 13%–79% increase in enzyme activity, with a trend toward greater increases in higher dose cohorts.^34^ There were serious adverse event (AEs) related to surgery in five patients: three intracranial hemorrhages (two asymptomatic),^31^, ^32^, ^34^ one venous hemorrhage,^33^ and one deep venous thrombosis and subsequent pulmonary embolus.^35^ A transient increase in dyskinesia was reported in eight participants.^34^, ^35^ One subject showed progression in disease and underwent DBS at 34 months post infusion.^35^

In 2018, AAV2‐AADC (VY‐AADC02, NCT03562494) was in phase II trial for people with advanced PD who were responding poorly to medications. The trial planned to recruit 85 patients and deliver 3.6 × 10^12^ vector genomes by a new delivery method via an occipital route and included control group receiving sham surgery. The primary outcomes safety measures, assessment of motor fluctuations at 1 year and putaminal coverage and enzyme activity. Changes on magnetic resonance imaging (MRI) resulted in FDA clinical hold on the trial in 2020. Subsequently Voyager therapeutics announced its partner Neurocrine Biosciences terminated the PD portion of their partnership ending the development of VY‐AADC.^36^

### Gene Therapy with Three Dopamine Synthesis Genes: *GTP cyclohydrolase I* , *tyrosine hydroxylase*, and *AADC*

This lentiviral gene therapy approach delivers three transgenes: tyrosine hydroxylase (*TH*), GTP cyclohydrolase I (*GCH1*), and aromatic L‐amino acid decarboxylase (*AADC*), transducing striatal neurons with the aim of increasing dopamine production (Fig. 1). There is one completed clinical trial involving 15 men who received lentivirus‐TH/GCH1/AADC through stereotactic intraputaminal bilateral infusions, in a phase I/II open‐label, dose‐escalation study,^28^ with subsequent long‐term follow‐up study of the same cohort (Table 1).^29^ The *off*‐state UPDRS‐III significantly improved at both 12 months (11.8 points)^28^ and long‐term follow up (8.2 points) without significant difference in dosage.^29^ There were no significant improvements in the *on*‐state UPDRS‐III. There was a 2‐point improvement in UPDRS‐IV at 12 months, but this was not sustained long‐term.^29^ UPDRS‐II (*off*‐state: 4 points; *on*‐state: 2 points) and PD Questionnaire (PDQ)‐39 (by 5.7 points) improved at 6 months,^28^ but again, this was not sustained long‐term.^29^ There were three serious AEs reported dyskinesia, acute psychosis, and a nervous system disorder (unspecified) in three different patients. The safety profile was similar across all dose cohorts with two deaths reported after 4 and 6 years, deemed unrelated to treatment. Eight patients required DBS surgical intervention after 2 years because of continued disease progression. Although the therapy appeared promising, concerns with long term durability and efficacy were partially attributed to deficits in delivery and vector design. This prompted development of OXB‐102, an optimized version with increased efficacy of dopamine production. The subsequent AXO‐Lenti‐PD study (NTC03720418) enrolled a higher dose cohort with higher delivery volume and flow rates and has reported significant improvements in UPDRS‐III *off* scores as well as improvement of *on* time at 6 months, when compared to baseline. Longer term study outcomes have not yet been reported.^37^ Unfortunately Sio Gene therapies returned the global rights for Axo‐Lenti‐PD to Oxford Biomedica in 2022 as they ceased their PD gene therapy program. This followed the resignation of the Sio CEO and indication of constraints of resources.^38^

Although these two approaches for targeted dopamine synthesis gene delivery are somewhat promising, the loss of dopamine in PD is the result of complex pathological processes that are likely to be multifactorial with genetic, epigenetic, and environmental influences. As such, supplementation of components of the dopamine synthesis machinery addresses only one aspect of the PD disease process and might not be sufficient to modify disease in the longer term.

Furthermore, developing gene therapy for PD poses several other scientific translational challenges. The pathogenic mechanisms governing PD remain incompletely understood and although there are transgenic and chemical animal models, none fully represent human pathology. Moreover, these models are not able to recapitulate the different stages of PD and little is known about how the disease state and cellular pathology impacts cellular uptake and efficacy of gene therapy. Clinical efficacy of these gene therapies may not be sustained as PD progresses^31^ and translating preclinical studies to estimate an efficacious human dosage at different disease stages is challenging—there are significant differences in diffusion, cellular transport, and cellular and extracellular architecture between species so a dosage/volume that is efficacious in a rodent model may only cover a fraction of the human target.^14^ Over time, clinical trials of AAV2‐AADC in PD have increased vector volumes, infusion rates, and vector genome dosages from 9 × 10^10^ vg in 200 μL initially to 4.7 × 10^12^ vg in 900 μL in the most recent trial, with the aim of increasing putaminal coverage.^30^, ^35^ The patients receiving higher dosages appear to show more sustained efficacy at 3 years.^35^ Interestingly, there are no significant differences in clinical efficacy between a mid‐range dose (1.5 × 10^12^ in 900 μL) and highest dosage, possibly suggesting a ceiling effect.^35^ It may be that with the infusion methodology, better transduction uniformity is not achieved with higher doses and the neurons close to the injection site are transduced by more virions.^14^ Longer term follow up studies will no doubt aid our understanding of the effect of dosage on long‐term clinical efficacy. It remains unclear whether treatment in much earlier disease stage to restore dopamine synthesis would have more meaningful sustained clinical impact and superiority over conventional treatment approaches. The difficulties are that this patient population is also likely to be those that would respond to drug treatments. Therefore, an invasive neurosurgical procedure to deliver viral gene therapy would seem unwarranted and the clinical trial results have not necessarily been shown to be superior or safer than as DBS surgery. The pathway to demonstrate safety, clinical efficacy, and improvement over currently available treatments have been very challenging in the PD viral gene therapy field and it would appear the interest from industry in dopamine synthesis gene therapy for PD is uncertain with the cessation of the Neurocrine Biosciences‐Voyager and Axo‐Lenti PD programs.

## Gene Therapy for Inherited Primary Neurotransmitter Diseases

### AADC Deficiency

AADC deficiency is an ultra‐rare, autosomal recessive neurodevelopmental disorder characterized by impaired monoamine synthesis of the catecholamines (dopamine, norepinephrine, and epinephrine) and serotonin. AADC deficiency patients have absent or non‐functional dopa decarboxylase (DDC) enzyme and therefore cannot convert l‐dopa to dopamine or 5 hydroxytryptophan to serotonin (Fig. 1).^39^ This disease, with a primary deficit in the AADC enzyme is very distinct to PD where there is gradual neurodegeneration and secondary loss of AADC enzyme. To date, neurodegeneration has not been reported in AADC deficiency and Ioflupane I 123 DaTScan are normal.^40^ Affected patients have a complex syndrome with motor, behavioral, neuropsychiatric, and autonomic symptoms. They typically present in infancy, with hypotonia, oculogyric crises (OGC), and developmental delay. The majority with classical early‐onset disease do not achieve head control although milder forms are reported, with a minority of patients achieving independent ambulation and spoken language.^39^ To date, over 135 patients have been described in the literature.^39^ Characteristically, most patients show limited benefit from standard drug treatments^39^ and there is significant morbidity and high risk of mortality.

Following demonstration of safety and early efficacy of intraputaminal delivery of rAAV2‐AADC gene therapy in PD, a logical application was to use this vector to treat children with inherited AADC deficiency. The first rAAV2‐AADC gene therapy study for AADC deficiency was a compassionate use trial in 2012 that has progressed to three further phase I/II clinical trials reporting long‐term efficacy in 2022 (Table 2).^41^, ^42^, ^43^, ^44^ Putaminal delivery directly transduces non‐dopaminergic neurons in the striatum; these include medium spiny neurons, bienzymatic non‐dopaminergic neurons that express TH and AADC, and monoenzymatic non‐dopaminergic neurons that express TH and are potentially able to produce dopamine.^30^ This approach has been used in three clinical trials treating 33 children with AAV2‐AADC through intraputaminal bilateral infusions (n = 26, one compassionate use study, one phase I open‐label, phase I/II open‐label studies) with follow‐up for 9 to 120 months.^41^, ^42^, ^43^, ^44^ All putaminally treated children reported clinical motor improvement, but continued to experience OGCs.^42^, ^43^ Five children that receive putaminal gene therapy in the compassionate trial have now been followed for 5 to 10 years.^44^ Of these, three remain stable in their Alberta Infant Motor Score (AIMS) and Peabody Developmental Motor Scores‐2 (PDMS‐2) assessments. One child had knee growth plate issues that impacted on assessment scores, possibly suggesting decline, but following orthopedic surgery, his motor function stabilized. Another patient showed decline in PDMS‐2 and AIMS scores at 5 years that was attributed to examination induced dystonia that was treated with aquatic therapy. His brain MRI was unremarkable with PET scan showing stable AADC expression and stable cerebrospinal fluid (CSF) homovanillic acid (HVA) levels.^44^ Four serious AEs were reported: one asymptomatic subdural hemorrhage related to the surgical procedure;^43^ CSF leak that required titanium mesh over the burr hole; one life‐ threatening hyperpyrexia^42^; and one transient increase of apneic episodes.^41^ Two deaths were reported because of Influenza B encephalitis.^42^

**TABLE 2:** rAAV2‐AADC clinical trials for AADC deficiency

Study	Participants	Age years	Follow up	Outcomes measures	Adverse events
Hwu et al.^41^ Compassionate use bilateral putaminal infusion	4 1.6 × 10^11^ vg‐320 μL	4.2–6.2	9–24	Safety and tolerability AIMS and PDMS‐2 motor score improvement in all patients CDIIT improvement in all patients Increased putaminal 18F‐DOPA uptake uptake in three patients	Transient choreic dyskinesia in all patients Transient increase in apneic episodes in one patient
Chien et al.^42^ Open‐label phase I bilateral putaminal infusion	14 1.8 × 10^11^ vg‐320 μL	1.7–8.4	24	Effect on motor development and CSF HVA and 5‐HIAA PDMS‐2 scores were increased (median: 62 points) HVA CSF concentration increased (median: 25 nmol/L) No significant change in 5‐HIAA CSF concentration	Transient dyskinesia in all patients (resolved with risperidone) 31 treatment‐related AEs, one severe (life‐threatening hyperpyrexia) 1 death due to Influenza B encephalitis, unrelated to treatment
Kojima et al.^43^ Open‐label/phase I/II bilateral putaminal infusion	6 2 × 10^11^ vg‐200 μL	4–19	24	Safety and tolerability AIMS motor scores improvement in all patients	Transient choreic dyskinesia in all patients 1 asymptomatic subdural hemorrhage related to surgery
Tai et al.^44^ Long term efficacy Compassionate and open‐label phase I/2 or 2b bilateral putaminal infusion	26 1.8 × 10^11^ vg‐320 μL (n = 21) 2.4 × 10^11^ 320 μL (n = 5)	1.7–8.5	12–120 months	AIMS and PDMS‐2 motor score improvement in all patients sustained for 12‐over 5 years follow up Increase in HVA CSF concentration No significant change in 5‐HIAA CSF concentration	CSF leak post‐surgery As previously reported in Hwu and Chien et al.^42^, ^43^
Pearson et al.^39^ Open‐label/phase dose escalation bilateral SN VTA infusion	7 8.3 × 10^11^ vg −160 μL (n = 3) 2.6 × 10^12^ vg‐160 μL (n = 4)	4.5–9	7–38	OGCs and GMFM‐88 improved in all patients Appearance of FMT‐PET uptake in all patients HVA CSF concentration increased in all patients (median 74 nmol at 6 months) No significant change in 5‐HIAA CSF concentration	21 AEs and 10 serious AEs reported Transient choreic dyskinesia in all patients Transient worsening of irritability and sleep disturbance 1 sudden death at home (attributable to the primary disease)
NCT02926066 Phase II bilateral putaminal infusion	To recruit 12 patients Jan 2022 commencement	2–6	12	Efficacy (changes in CSF neurotransmitter concentrations and PDMS‐II) at12 months Safety and AEs, pharmacokinetics, changes in FDOPA‐PET scan.	No data available yet
NCT01395641 Phase I/II bilateral putaminal infusion	To recruit 10 patients Jan 2022 commencement	Over 2 years	12	Efficacy (changes in CSF neurotransmitter concentrations and PDMS‐II) at 13 months Safety and AEs, pharmacokinetics, changes in FDOPA‐PET scan. Safety and AEs, pharmacokinetics, changes in FDOPA‐PET scan	No data available yet

Abbreviations: 5‐ HIAA, 5 hydroxyindoleacetic acid; AEs, adverse events; AIMS, alberta infant motor scale; CDIIT, comprehensive developmental inventory for infants and toddlers; CSF, cerebrospinal fluid; GMFM 88, gross motor function measure; HVA, homovanillic acid; OGCs, occulogyric crisis; PDMS ‐2, peabody developmental motor scale.

An alternative approach for AADC deficiency has also been developed, whereby rAAV2‐AADC is delivered to target the SN and ventral tegmental area (VTA) by convection‐enhanced midbrain stereotactic injection.^40^ This approach aims to transduce the midbrain and harness anterograde axonal transport properties of rAAV2 to deliver AAV2‐hAADC to striatonigral network. Seven children, ages 4 to 9 years underwent convection‐enhanced delivery of AAV2‐hAADC in two dose cohorts: 1.3 × 10^11^ vg (n = 3), and 4.2 × 10^11^ vg (n = 4) achieving target coverage of 98% and 70% of the SN and VTA, respectively.^38^ OGCs resolved in six of seven children by 3 months post gene therapy and six children gained normal head control and 4/7 could sit independently by 12 months. At 18 months, two subjects were walking with support.^38^ CSF HVA increased significantly 3 months after gene delivery and was sustained.^38^ There was one unexpected sudden death at 7 months post gene therapy in one child that was attributed to underlying disease (and not treatment related). The subject had shown positive improvements up to 6 months assessments post gene transfer.^40^

Across the different studies, the greatest improvements appear to be in participants with mild to moderate disease presentation with rapid improvements within 12 months post treatment.^40^, ^41^, ^42^, ^43^, ^44^ Both midbrain and putaminal delivery report an amelioration in the AIMS, PDMS‐2, or Gross Motor Function Measure‐88 (GMFM‐88).^40^, ^41^, ^42^, ^43^, ^44^ There was a trend toward greater improvement in younger participants, although longer term studies will better inform on this early observation. Dystonic attacks improved and OGCs decreased in frequency and/or severity to varying degrees, but amelioration of OGCs was reported more specifically with midbrain delivery.^40^ Swallowing and respiratory symptoms were improved in all treated children. Cognitive assessments improved in all participants (Kyoto Scale of Psychological Development‐Cognitive‐Adaptation and Language‐Sociality, or Comprehensive Developmental Inventory for Infants and Toddlers). Qualitative improvements in feeding, mood, autonomic function, and sleep were also reported by caregivers.^40^, ^41^, ^42^, ^43^ Putaminal (FMT‐PET)^41^ and midbrain, putaminal, and caudate uptake (18F‐DOPA PET) improved from baseline absence to bilateral high signal intensity (indicating restoration of AADC activity), up to 5 years post gene therapy.^44^ CSF neurotransmitter analysis showed significantly increased or a trend toward increased dopamine metabolites from baseline, although levels remained below normal reference ranges in all studies on CSF sampling at 6–months.^40^, ^41^, ^42^, ^43^ The change in serotonin metabolites varied and did not show significant increase irrespective of delivery target.^40^, ^41^, ^42^, ^43^, ^44^ All participants experienced transient dyskinesias and choreiform movements for weeks to months after gene therapy, but these were manageable with medication adjustments.^40^, ^41^, ^42^, ^43^

Overall, there is now a growing body of evidence to suggest that gene therapy can potentially ameliorate dopamine synthesis, neurotransmission, and homeostasis in AADC deficiency. As described, this manifests with improvement of the core disease phenotype, with motor gains and neurodevelopmental progress. The mechanisms underpinning the neurodevelopmental effects are not clear, but studies in a patient‐derived induced pluripotent stem cell (iPSC) neuronal model treated with LV‐hAADC showed that an increase in AADC protein levels and restoration of enzyme activity was associated with a significant increase in synaptophysin protein and primary neurite branching.^45^

Recently, the EMA and MHRA have granted authorization for the use of eladocagene exuparvovec (Upstaza) for AADC deficiency patients by targeted putaminal delivery. It is too early to evaluate the differences in efficacy between the intraputaminal and midbrain targeted delivery approaches. The midbrain target is smaller in volume than the putamen, but higher dosages (8.3 × 10^11^ to 2.6 × 10^12^ vg)^40^ were delivered to the midbrain when compared to the putamen (1.8 × 10^11^ to 2.4 × 10^11^ vg).^41^, ^42^ Neuronal dopamine synthesis and metabolism is highly regulated^46^ (Fig. 1) and in dopamine normostasis, the key enzymes are closely regulated to prevent excessive oxidative stress from dopamine and DOPA oxidation.^46^ It is unclear if midbrain delivery of AAV2‐AADC may provide a more physiological restoration of AADC into dopaminergic neurons, where the full complement of dopaminergic enzymes and transporters are present. Theoretically, midbrain delivery may potentially reduce the risk of dopamine‐related oxidative stress. Longer term data on patients treated by both midbrain and putaminal gene delivery will inform on the relative merits and strengths of the different brain delivery approaches.

Finally, an important future consideration is that AADC enzyme is also key for serotonin biosynthesis and affected patients show brain serotonin deficiency.^39^ Neither of the gene therapy delivery approaches has targeted serotonergic neurons and unsurprisingly, no effect on CSF serotonin metabolites has been observed.^40^, ^41^, ^42^, ^43^, ^44^ The clinical manifestations of residual serotonin deficiency in these patients are yet to be fully understood. This is no doubt an area for future therapeutic development as potentially dual delivery approaches targeting both the basal ganglia (putamen/midbrain) and brainstem will need to be considered for next generation gene therapies.

### DTDS

The preclinical and clinical data on rAAV2‐AADC gene therapy for PD and AADC deficiency have inspired our own efforts to develop gene therapy for DTDS. This is an ultra‐rare inherited disorder caused by biallelic loss‐of‐function mutations in *SLC6A3* encoding the dopamine transporter (DAT).^47^, ^48^, ^49^ DAT regulates dopamine reuptake into the presynaptic neuron to terminate dopamine neurotransmission and is, therefore, a key transporter controlling dopamine homeostasis. Affected infants present with hyperkinesia, dystonia, and chorea and then later develop parkinsonian features, with bradykinesia, rigidity, and tremor.^47^, ^48^ They do not respond to pharmacotherapies or DBS and therefore, gene therapy is potentially a suitable approach to address the underlying etiology.

Proof of concept gene therapy was first performed in the DAT knockout model by *Illiano* and colleagues^50^ using two AAV vectors, of capsid serotype 10, delivered into the midbrain of adult DAT mice by stereotactic injection. To achieve high specificity for dopaminergic neurons, the first AAV expressed Cre recombinase under the control of the truncated rat TH promoter and a second AAV contained murine DAT flanked by loxP sites, under the control of a constitutive Cytomegalovirus (CMV) promoter. Although results were encouraging with amelioration of motor phenotype, the use of mouse *SLC6A3* cDNA and co‐expression with Cre recombinase limited the potential of this approach for clinical translation.

We aimed to develop a more directly translatable approach. We generated initial proof of principle using a DTDS patient‐derived midbrain dopaminergic model.^51^ Patient‐derived neurons showed impaired DAT activity and disease‐specific apoptotic neurodegeneration.^51^ To evaluate gene therapy in vitro, we generated a LV‐hSLC6A3 construct for transduction at day 24 differentiation and analysis at day 65 of derived maturity. Lentiviral gene therapy led to restoration of DA uptake and restored neuronal survival with no evidence of neurodegeneration.^51^ We, then, used the DAT knockout mouse model to progress viral hSLC6A3 gene therapy to clinical application. A neonatal gene therapy study with rAAV9‐hSyn.hSLC6A3 in the DAT knockout was undertaken, as targeted stereotactic injections are not feasible in neonatal mice. P0 pups were treated with intracerebroventricular rAAV9‐hSLC6A3 gene therapy transducing the whole brain. This rescued the DAT knockout mouse (DAT KO) model restoring normal survival and motor behavior, but off‐target expression in the prefrontal cortex showed neuronal loss and an astroglial inflammatory response.^51^ To restrict expression to target SN and VTA where DAT is expressed, we then delivered rAAV2‐SLC6A3 by stereotactic midbrain injection, directly modelling future clinical application in a two log dose ranging study. Here, survival and locomotor activity were fully rescued in the KO model. There was sustained DAT expression in the midbrain with anterograde transport of AAV2‐SLC6A3 resulting in striatal expression of high dose treated animals with absence of neuropathology.^51^ Our data demonstrated that stereotactic midbrain delivered rAAV2‐SLC6A3 gene therapy provides correction of DAT function with clear safety in the mouse model of DTDS, thereby accelerating the route to clinic.

### Future Directions

Translating gene therapy to optimize efficacy in the clinic is an iterative process. rAAV2‐AADC vector has been studied in 10 clinical trials in PD and AADC deficiency over 13 years with continual modification of delivery methods, infusion volumes and rates, image guidance, and vector dosage. Interestingly, no modifications on the vector design have been made.

Gene therapy for dopaminergic disorders involves complex challenges to target specific brain regions and cell types. Advances in viral vector technology to improve dopaminergic selectivity have been focused on AAV capsid and promoter development.^9^ Approaches are increasingly sophisticated and may overcome some of the current hurdles experienced. A key challenge is achieving transgene expression at physiological levels in the target cell type. To progress gene therapies, we must gather knowledge on how gene and protein expression levels change in normal neurodevelopment as endogenous levels may vary at different developmental time points. This may be critical in tailoring gene therapies for sustained efficacy in the lifetime of the developing child. Furthermore, gene expression levels may vary with disease stage and therefore, there is need to develop gene therapies that are responsive to this, for example, addressing the declining levels of AADC enzyme or neurotrophic factor in the progressive neurodegeneration of PD.

Two newer preclinical rAAV‐AADC gene therapies have been developed, providing wider transduction of neurons in the SN, VTA, and dorsal raphe.^52^, ^53^ In an earlier study, P0 neonatal AADC knockin Ddc^KI^ mice received bilateral intracerebroventricular injections (total 4 × 10^10^ vg per pup) of rAAV9.CMV.hAADC.^52^ The treated mice showed higher body weights with 90% survival compared to 65% survival in untreated Ddc^KI^ mice.^52^ Gene therapy ameliorated hindlimb clasping and cardiovascular abnormalities. Supraphysiological levels of AADC enzyme were detected at twofold of wild‐type levels. Dopamine levels significantly increased (equivalent to wild‐type), whereas serotonin and 3‐O‐methyldopa improved, but were not restored to wild‐type levels. Treated Ddc^KI^ mice were more hyperactive and widespread AADC expression throughout the brain in neurons and astrocytes was observed.^52^ Although therapeutic efficacy was demonstrated, the off‐target expression could result in ectopic dopamine synthesis, possibly accounting for the behavioral hyperactivity.^52^ To improve selective neuronal expression, a tyrosine‐mutant pseudotype AAV9/3 vector containing a synapsin promoter expressing mouse AADC with WPRE (AAVN‐AADC) was evaluated.^51^ In this study, P7 Ddc^KI^ received systemic gene therapy by intraperitoneal injection 4.6 × 10^11^ vg AAVN‐mAADC or AAV9‐CMV‐hAADC compared to untreated Ddc^KI^ and wild‐type mice. From P21, both treatment groups showed higher body weights with improved survival of 95% and 78%, respectively.^53^ Systemic gene therapy rescued behavioral phenotype with higher brain dopamine and serotonin achieved with AAVN‐mAADC. The hyperactivity observed with AAV9‐hAADC was not seen in AAVN‐mAADC treated Ddc^KI^.^53^ This vector resulted in selective neuronal expression of AADC, but intraperitoneal delivery resulted in off‐target liver expression (although no hepatoxicity or immune response was observed in the mice).^53^ These studies provide important proof‐of‐concept that (1) wider brain expression of AADC is associated with not only increased dopamine levels, but also increased brain serotonin and (2) the observed off‐target effects with non‐targeted delivery approaches pose safety concerns and need to be avoided. Transduction of serotonergic dorsal raphe nuclei should be considered, as previously discussed, but is a highly challenging neurosurgical target within the brainstem. This preclinical study demonstrates proof of concept approach to improve on‐target expression and safety using novel capsid and neuronal selective promoter. The AAV2‐AADC vectors using in PD clinical trials and Upstaza eladocagene exuparvovec are under the transcriptional control of a ubiquitous promoter (CMV). The current AAV vectors do not express selectively for specific cell types and we have approached improving on‐target expression with use of neuronal promoter and AAV2 capsid stereotactic delivery. There is preclinical development of small cell‐type specific promoters that may improve expression efficiency and safety by restricting expression to disease target cells underway.^54^

AAV capsid engineering has evolved over 2 to 3 decades in two main ways: directed evolution and rational design. In directed evolution systems, a random process to shuffle the capsid gene is applied to known serotypes (such as peptide insertions into known sites of AAV capsid or phage display). Rational design to refine capsid structure to achieve desired characteristics, such as disruption of native cellular binding motifs and insertion of high affinity ligands in the Cap gene, is an alternative strategy. Interestingly, few synthetic capsids have replaced wild‐type variants in preclinical application and none have reached clinical trial, because the improved novel feature is seldom transferred from host species (most commonly screened initially in mouse) to non‐human primate or humans. There have been several efforts to generate synthetic capsids for PD with superior transduction efficacy for the striatonigral pathway.^55^ Through rational design using the BRAVE system, tropism screening has been performed with HEK293T cells, primary cortical neurons, rats transplanted with DA neurons derived from human embryonic stem cells, and human iPSC derived organoids.^9^ Through this strategy, 25 synthetic capsids with superior properties over wild‐type AAV2 have been identified, including the MNM008 capsid with increased retrograde infectivity of rat and human dopaminergic neurons.^9^ It is conceivable that such superior AAV capsids will have an important role in the future development of gene therapies for disorders of the dopaminergic system.

## Conclusions

Over time, there is increasing evidence of the efficacy of dopaminergic gene therapy for movement disorders associated with dopamine dyshomeostasis. The most compelling data stems from the rAAV2‐AADC gene therapy approaches for AADC deficiency. Gene therapy approaches for such monogenic disorders have the potential to be significantly impactful. Trials in PD and AADC deficiency suggest that the disease stage could impact clinical outcome, emphasizing the importance of the therapeutic window. There are many challenges to address in the development of gene therapy for dopaminergic diseases both at the preclinical and clinical level; these include improving delivery, distribution, control of gene expression and improving preclinical modelling of dopaminergic disorders. Future directions to develop next generation gene therapies include design of novel dopaminergic specific capsids and promoters to tailor gene therapy for these disorders improving gene expression in target diseased cells and minimize off‐target effects. Clinically impactful genetic therapies for PD will need more sophisticated approaches to address the complexity of neurodegenerative disorders. The strategy of viral mediated dopamine enzyme replacement is only one of many novel therapeutic approaches. Strategies to halt or ameliorate aberrant α‐synuclein folding, improve mitochondrial function, restore dopamine synthesis, improve synaptic dysfunction, improve neuronal survival, or cell replacement therapies are future strategies and potential combined approaches may lead to the first steps toward halting PD disease progression. In the longer term, future scientific discoveries will better define etiologies and PD pathomechanism, to improve early diagnosis, identify clinical therapeutic window, and potential therapeutic targets to enable treatments that may reverse disease.

## Author Roles

(1) Research Project: A. Conception, B. Organization, C. Execution; (2) Statistical Analysis: A. Design, B. Execution, C. Review and Critique; (3) Manuscript Preparation: A. Writing the First Draft, B. Review and Critique.

J.N.: 3A, 3B.

S.B.: 3A, 3B.

S.NW.: 3A, 3B.

M.A.K.: 3A, 3B.

## Financial Disclosures

J.N., M.A.K., and S.N.W. are co‐inventors on patent application titled Gene Therapy for DTDS (GB2101958.3). M.A.K. was sponsored by Agilis to attend the AADC Deficiency International Advisory Board (AADC‐D IAB) on June 27, 2018. S.N.W. has previous or existing consultancy agreements with ONO Pharmaceuticals, Synpromics, Reliance Biosciences, Codiak Biosciences, Takeda Pharmaceutical Company, and LivaNova Plc. M.A.K. and S.N.W. hold or have previously held consultancy agreements with Biomarin, and S.N.W. and J.N. also have consultancy agreements with Albion Capital. J.N. and S.N.W. have sponsored research agreements with Synpromics/Askbio Europe and Rocket Pharma. M.A.K and S.N.W. are founders of Bloomsbury Genetic Therapies. J.N. holds equity in Bloomsbury Genetic Therapies.

## Data Availability

Data sharing not applicable to this article as no datasets were generated or analysed during the current study.
